# Current diagnostic and quantitative techniques in the field of lymphedema management: a critical review

**DOI:** 10.1007/s12032-024-02472-9

**Published:** 2024-09-05

**Authors:** Mary Vargo, Melissa Aldrich, Paula Donahue, Emily Iker, Louise Koelmeyer, Rachelle Crescenzi, Andrea Cheville

**Affiliations:** 1 Department of Physical Medicine and Rehabilitation, MetroHealth Rehabilitation Institute, Case Western Reserve University, Cleveland, OH USA; 2 Center for Molecular Imaging, UTHealth, Houston, TX USA; 3 Department of Physical Medicine and Rehabilitation, Vanderbilt University Medical Center, Nashville, TN USA; 4 Lymphedema Center, Santa Monica, CA USA; 5 Australian Lymphoedema Education, Research & Treatment (ALERT) Program, Macquarie University, Sydney, Australia; 6 Department of Radiology and Radiological Sciences, Vanderbilt University Medical Center, Nashville, TN USA; 7 Department of Physical Medicine and Rehabilitation, Mayo Clinic, Rochester, MN USA

**Keywords:** Lymphedema, Diagnosis, Screening, Monitoring, Measurement, Imaging, Cancer

## Abstract

Lymphedema evaluation entails multifaceted considerations for which options continue to evolve and emerge. This paper provides a critical review of the current status of diagnostic and quantitative measures for lymphedema, from traditional and novel bedside assessment tools for volumetric and fluid assessment, to advanced imaging modalities. Modalities are contrasted with regard to empirical support and feasibility of clinical implementation. The manuscript proposes a grid framework for comparing the ability of each modality to quantify specific lymphedema characteristics, including distribution, dysmorphism, tissue composition and fluid content, lymphatic anatomy and function, metaplasia, clinical symptoms, and quality of life and function. This review additionally applies a similar framework approach to consider how well assessment tools support important clinical needs, including: (1) screening, (2) diagnosis and differential diagnosis, (3) individualization of treatment, and (4) monitoring treatment response. The framework highlights which clinical needs are served by an abundance of assessment tools and identifies others that have problematically few. The framework clarifies which tools have greater or lesser empirical support. The framework is designed to assist stakeholders in selecting appropriate diagnostic and surveillance modalities, gauging levels of confidence when applying tools to specific clinical needs, elucidating overarching patterns of diagnostic and quantitative strengths and weaknesses, and informing future investigation.

## Introduction

Lymphedema diagnostic methods comprise an area of vigorous current research, with multifaceted priorities including early detection, establishing diagnosis and pathophysiology, localization, guiding intervention, and surveillance of severity and response to therapy [1]. Clinical use requires weighing empirical standards including favorable reliability, validity, diagnostic accuracy, precision, and sensitivity, as well as meeting feasibility demands related to cost, time, space, level of training, level of personnel, least possible risk, and patient acceptance. In the setting of cancer-related lymphedema, increasing rigor continues to be applied to a prospective surveillance and early intervention model of care. Additionally, in patients with cancer diagnoses, differential diagnostic distinctions may arise between benign versus malignant lymphedema, between lymphedema and neuromusculoskeletal or soft tissue conditions producing confounding locoregional symptoms, and between lymphedema and other etiologies of limb swelling or enlargement, especially venous disorders, lipedema, and systemic comorbidities.

Most of the existing diagnostic literature for lymphedema emphasizes breast cancer-related and upper quadrant lymphedema, especially regarding traditional or common measures such as tape measurement, water displacement and bioimpedance spectroscopy [2]. Lower extremity lymphovenous insufficiency is also extremely common, yet not as well studied [3]. Given the importance of lymphedema in the vascular space, three American vascular societies collectively published an expert opinion consensus on lymphedema diagnosis and treatment which suggests agreement from the experts with diagnostic work-up and risk factors for lymphedema, though highlights the high degree of variability regarding lymphedema treatment and practice patterns necessitating further investigation [4]. Furthermore, the International Society of Lymphology 2020 guidelines highlight the complexity and vast array of lymphedema clinical contexts, which “impact individualized patient care” [5]. Additionally, lymphedema has a striking 90% incidence in the survivors of head and neck cancer, however is still drastically under-diagnosed and treated from a recent publication utilizing a large US healthcare claims repository database [6]. Thus, there is a critical need for understanding and incorporating diagnostic and quantitative techniques into routine clinical practice which spans a significant and diverse population of individuals living with lymphedema.

Regarding detection protocols, overriding themes which have emerged in the literature include the importance of obtaining a baseline preoperative or pretreatment measurement whenever possible [4, 7–9], and also to employ a consistent measurement modality in surveillance over time, rather than using different methodologies interchangeably [2, 10]. None of the diagnostic methods have perfect accuracy and therefore there can be value in clustering observations from different available perspectives [2]. A 2020 systematic review of guidelines for detection of lymphedema noted overall limited evidence quality and could not make definitive conclusions, but found common themes including importance of clinical evaluation, use of duplex ultrasound to assess tissue thickness and fibrosis, computed tomography (CT) or magnetic resonance imaging (MRI) to rule out neoplasm, lymphoscintigraphy when needed to establish diagnosis, and measurement strategies including circumferences, perometry, and bioimpedance [3]. There is increasing advocacy for universal guidelines, especially for breast cancer-related lymphedema [11], incorporating symptoms, clinical examination and objective measurement, and also that all at-risk patients receive regular screening and education [12].

This paper explores the spectrum of traditional through emerging modalities, with the goal of evaluating practicable strategies by type of clinical scenario. The emphasis is on peripheral lymphedema, especially cancer-related lymphedema. Aims include (1) facilitation of best decision-making for modality choice in clinical and research applications, and (2) allowing overarching patterns to be identified, including strengths and gaps in current literature with regard to identified lymphedema assessment priorities.

## Methods

A critical review was conducted by an experienced group of clinicians and researchers in the field of lymphedema and cancer rehabilitation, who identified modalities utilized for lymphedema diagnosis and analysis, and also identified core dimensions or purposes of lymphedema evaluation. The group was comprised of three physiatrists (AC, EI, MV), one physical therapist (PD), one occupational therapist (LK), one immunologist (MA), and one biochemist/molecular biophysicist (RC). The work of the group was conducted over a one-year period including independent efforts, six virtual meetings, and interspersed electronic communications. Preliminary content was presented at the Lymphedema Summit, sponsored by the Lymphology Association of North America (LANA) and the American Cancer Society (ACS), on October 7, 2023. Virtual meetings focused on the format and focus of this work, and, especially, on consensus with regard to the modalities themselves and on the integrative frameworks for core dimensions of lymphedema evaluation.

Via standard literature review, each of the modalities was briefly summarized with regard to their major capabilities and limitations, empirical and feasibility characteristics, as well as clinical versus research applicability. Primary authorship of each modality section was accorded with consideration of the diverse expertise of the authors, for content areas including history and physical examination (MV), tape measurement (MV), water displacement (MV), perometry (PD), bioimpedance (LK), tissue dielectric constant (PD), tonometry (PD), three-dimensional imaging (LK), patient reported outcomes (AC), lymphoscintigraphy (EI), ultrasound (EI), CT (EI), near-infrared fluorescence (MA), and MRI (RC). These summaries incorporated literature search as well as the expertise and experience of the authors. The literature search was broad and included primary studies as well as existing summative materials such as reviews and practice guidelines. An electronic mailbox was kept to which all authors had access, which included these documents, and other resources such as images and presentation materials. Each of the summaries was reviewed by all of the other authors and areas of controversy resolved via group communication.

Subsequently, via expert consensus process, five separate grids were generated with regards to the utility of each modality for assessing lymphedema characteristics, as well as for clinical dimensions of screening, diagnosis, individualizing treatment, and monitoring treatment response. Within each of these five categories, critical subcategories were identified through group consensus, and the utility of each modality for each subcategory was assigned a score of strong (S), indeterminate (I), unknown (U) and not useful or not applicable (N). A strong (S) rating was applied when the modality has an excellent favorable evidence basis as pertains to the specific subdomain, or, alternatively, a “Strong” rating could also be applied when at least 3 of 4 of the following four criteria were met:

- Clinical practice-based acceptance, track record.
- Some published evidence to support use.
- Expert consensus.
- Compelling theoretical basis.

If less than three of the above criteria were met but there remained a compelling rationale for use, an “intermediate” (I) rating would be assigned, such that at least 2 criteria were met or one criterion was strongly met. Feasibility was not used as a separate criterion, as this can vary with availability and accessibility of diagnostic modalities in individual treatment settings. However, feasibility considerations are generally implied within the clinical practice-based acceptance criterion. In situations of contextual variability, interpretation leaned towards the contemporary application of the modality (e.g., bioimpedance for early detection of breast cancer-related lymphedema). Furthermore, areas were highlighted that are especially relevant for research applications or a particularly active focus of current investigation.

The noninvasive office-based measures, including history and physical (H&P), patient reported outcome measures (PROMs), tape measure, perometry, water displacement, bioimpedance, tissue dielectric constant (TDC), tonometry, and three-dimensional (3D) camera, were placed on the left side of the charts and the more advanced imaging measures, including lymphoscintigraphy, ultrasound (US), indocyanine green lymphography (ICG), CT, and MRI, on the right side of the charts. It is recognized that ICG and ultrasound may also be performed in office, however they were considered advanced for the purpose of this review given their current less-frequent application by direct-treating lymphedema clinicians.

## Results

### Lymphedema Assessment Modalities at Bedside or Office

#### History and physical examination

Clinical H&P encompasses numerous domains including symptoms, time course, underlying risk factors, such as infectious history and other medical comorbidities, mobility, positioning, family and social history, cutaneous and soft tissue effects, motor-sensory and musculoskeletal abnormalities, and body weight. A varied taxonomy of descriptive terms may apply to skin and soft tissue characteristics, including basic characteristics as pitting, firmness, fibrosis, thickening, or erythema, to more elaborate descriptors including, but not limited to hyperkeratosis, lipodermatosclerosis, papillomatosis, nodular fibrosis, lymphedema rubra, phlebolymphedema, lymphangiomas (blisters containing lymph fluid), sausage digits, and elephantiasis verrucosa nostra [13–16]. Associated observations may include evidence of cellulitis, lymphorrhea, or wounds [5]. Palpation is employed to assess fibrosis, including skin and deeper tissue mobility, and extent of pitting, but reliable grading criteria for fibrosis are needed [17]. In the lower extremities, Stemmer’s sign (inability to pinch a skinfold at the dorsum of the second toe) is a classic observation [13, 18], and individuals with history of axillary lymph node dissection may exhibit axillary cording especially early in the posttreatment course.

Various staging or grading paradigms exist, most notably the International society of lymphology (ISL) system ranging from subclinical lymphedema to lymphostatic elephantiasis, and which contains morphologic as well as size criteria [5, 19]. The ISL stages include subclinical (0 or Ia), early accumulation with pitting (I), increased fat and fibrosis infiltration (II) and lymphostatic elephantiasis (III). Other paradigms have also been employed. The Campisi scale delineates 5 stages including, in order of severity, initial/irregular edema, persistent lymphedema, persistent lymphedema with lymphangitis, fibrolymphedema, and elephantiasis [13, 20]. The National Cancer Institute-Common Terminology Criteria for Adverse Events (NCI-CTCAE) 5.0 (Grades 1–3), emphasizes soft tissue and skin characteristics, and in the severe category this scale incorporates limitation of self care as a criterion [21]. An earlier version, NCI-CTCAE 3.0 incorporates multiple lymphedema-related contexts including limb, head and neck, trunk/genital, lymphocele, cording and lymphedema-related fibrosis [22]. While not specific to lymphedema, the Pitting Edema Scale (1–4) has also been applied to lymphedema evaluation though reliability is likely limited [23].

Despite its ubiquity and importance to clinical care, studies are lacking with regard to accuracy and intra/interrater reliability of the physical examination for either clinical or research settings [4]. Lymphedema staging has value as a conceptual shorthand, and high feasibility to be incorporated into office visits. Limitations of clinical staging include imprecision regarding severity, that features of more than one stage may be present in the same individual, and that mixed etiologies of swelling may be present.

#### Bedside and office measures: general statement

An understanding of how to use and interpret the results of the bedside or office-based tools for surveillance and monitoring can greatly enhance the earliest diagnosis and treatment of lymphedema as well as give objective measures of change related to therapeutic intervention. A word of caution: no one device to-date can adequately measure all elements of therapeutic change given the heterogeneity of lymphedema. Furthermore, utility may break down in the absence of a reliable benchmark for comparison, such as a baseline measurement or unaffected contralateral limb. This requires a clinician to contextualize clinical interpretation.

#### Volume measurement

Volume measurement is an extension of the physical examination that can be undertaken with varying levels of technological sophistication. While parameters for detection exist, none are universally agreed upon, but volumetric thresholds of 3%, 5% or 10% change from baseline or in comparison to a normal contralateral side have been employed [5, 24], as well as absolute value of 150-200 ml difference in volume [25]. ISL 2020 guidelines define severity as minimal (> 5 < 20% increase in limb volume), moderate (20–40% increase), or severe (> 40% increase), with some clinics preferring to use > 5–10% as minimal and > 10– < 20% as mild. The variability in limb volumes between different individuals limits utility of using absolute volume change as a severity parameter for breast cancer-related lymphedema [24, 26]. Good reliability and validity have been found with numerous techniques including the traditional objective measures of water volumetry and tape measurement, as well as with more advanced approaches including perometry and (the nonvolumetric technique of) bioimpedance spectroscopy [27]. While most research has been conducted for breast cancer-related lymphedema, these principles apply to all types of lymphedema, including patients at risk for secondary lymphedema of the lower limbs who have had central operations with nodal sampling or resection.

#### Tape measurement

Tape measure-derived circumferential and volumetric measurements remain the most common clinical method [5, 10, 13, 28, 29] as well as the most extensively researched with regard to its reliability, validity and diagnostic accuracy [2, 27, 30, 31]. Tape measurement has been employed in multiple trials, but there is potential for interrater variability, and likely low sensitivity for subclinical disease [24].

A difference or change of 2 cm or more at any one measurement site has been described as significant, (though of questionable reliability, especially in the setting of higher limb girths)[19] versus a combined difference of 5 cm or more summed over 5 sites along the limb [17]. Volumetric conversions may be performed by fulstrum or truncated cone calculations, with the former considered more accurate and less likely to overestimate volumes [31]. Formulaic adjustments also exist which account for dynamic factors such as body weight [32].

There is not universal agreement on measurement paradigms between fixed intervals or landmark-based. Taylor et al., examining the arms of breast cancer survivors with lymphedema, found volumes calculated from anatomic landmarks to be reliable, valid, and more accurate than those obtained from circumferential measurements based on distance from fingertips [30]. Sun et al. concurred with these findings, in a volumetric study of breast cancer patients assessed serially postoperatively, comparing landmark versus fixed-interval derived tape measurements to perometry as the gold standard. In the Sun study, the landmark-based measurements had better sensitivity and specificity than the fixed interval measures (93.1%/63.1% overall for landmark-based and 81.9%/16% for 4 cm intervals) though the landmark technique underestimated upper arm volumes and had only 63.2–66.7% sensitivity for the lower (5–10%) relative volume change subcategory [33].

Tape measurement and water displacement are highly correlated but can yield different values, with between 5% and 15–19% variation reported, so these techniques cannot be used interchangeably [30, 31]. While reported diagnostic thresholds for treatment initiation vary, a clinical practice guideline on upper quadrant lymphedema yielded Grade B recommendations for tape measure use including volume ratio of 1.04 from the unaffected limb, volume differential > / = 200 ml between sides, and 5% or greater increase from baseline, but recommended against using a single site 2 cm difference as a diagnostic criterion [2].

Tape measurement has excellent feasibility regarding expense and accessibility. Concerns include the need for standardized protocols, training, and time to complete and compute measurements. Tape measurement is most applicable for limb rather than axial lymphedema, and like most volumetric measures it does not capture tissue composition.

#### Water displacement

Water displacement tanks for volumetric assessment have a limited role in most clinical settings due to feasibility barriers, though can be advantageous in research settings due to high degree of precision, with upper limb standard error of measurement of 3.6% compared to 6.6% for tape measurement [27]. However, error may be introduced if the limb is not submerged to a consistent level [30].

While water displacement is valid, accurate and reliable, disadvantages include being cumbersome, non-portable, requiring space, time and with risk of cross contamination [10, 30, 34]. Additionally, water displacement indicates the totality of volume but does not provide geometric detail on focal areas of enlargement. Measurement of lymphedema involvement near the root of the limb may be limited [5]. The Clinical Practice Guideline developed by the Oncology Section of the American Physical Therapy Association gave water displacement a Grade B recommendation, with criteria of > 200 ml or > 10% interlimb difference [2].

#### Perometry

Perometry provides a quick measure of limb circumference measures and calculated summed volume using an infrared scanner frame that slides over the body region in seconds. The device measures arms, legs, torso, hands and feet depending on the perometer model. The advantages of using perometry are reliability of repeat measurements, rapid measurements with ease of repeating measurements and ability to evaluate limb circumference at 0.5 cm increments. Additionally, measures can be used for compression garment fitting, though some additional measurements may be necessary with custom garment fitting. In breast cancer-related lymphedema, perometry has good correlation with circumferential measures and good correction with bioimpedance spectroscopy [35, 36]. Disadvantages include potential awkward patient positioning, expense of device, not easily transported requiring designated space, and limited centers with access to the equipment mostly found in research centers [35, 37]. Additionally, there is difficulty with hand and foot measurements and positioning can be problematic for some patients to get accurate reproducible arm and leg volume results where consistency in measurement technique needs to be maintained for repeated measures over time [35, 38]. Although considered a reliable measure of limb size and offers potential earlier detection of swelling compared to tape measure and palpation, as with other volume and circumference measurements, perometry is unable to discern extracellular fluid accumulation directly, rather volume changes could be related to muscle and fat changes as well within the area of interest. Therefore, similar to other devices of water displacement and tape measurements, perometry is not recommended as the sole method to measure and monitor lymphedema.

#### Bioimpedance

Bioimpedance technology is a method that measures biological impedance at different frequencies permitting clinicians to assess fluid compartments and body composition. This potentially provides clinicians a variety of information to help with making a clinical diagnosis of lymphedema and tailoring patient care treatment. Multiple frequency bioelectrical impedance analysis (MFBIA) and bioimpedance spectroscopy (BIS) provide a more comprehensive analysis compared with single frequency bioelectrical impedance analysis (SFBIA) by obtaining bioimpedance data at several different frequencies (MFBIA at different points from 1 to 1000 kHz and BIS continually from 0 to 1000 kHz) where BIS includes the crucial 0 kHz frequency [39] as part of its assessment. Impedance comprises resistance and reactance, indicating opposition from body fluids and cell membranes, respectively [40]. The inverse relationship between impedance and tissue fluid volume is a key principle [41]. The frequency of the current determines what is being measured, with zero frequency current unable to penetrate cell membranes [42]. Additionally, bioimpedance measures provide body composition data and phase angle data where a growing body of research is promising to inform oncology clinicians on changes to lean body mass and a prognostic factor in the advanced cancer setting, respectively [43, 44].

Bioimpedance devices gage resistance to electrical current flow, especially in the extracellular fluid compartment at low frequencies. More specifically for bioimpedance spectroscopy (BIS) devices, an “impedance ratio” methodology is utilized for assessing unilateral arm or leglymphedema. This involves comparing the resistance at 0 kHz in the affected/at-risk limb to that in the unaffected limb, expressed as a ratio [45]. Alternatively, this ratio can be linearized into an L-Dex score, where abnormal values indicate deviations from the normal range (− 10 to + 10 L-Dex units) and a change exceeding 6.5 L-Dex units from baseline signifies subclinical lymphedema [46]. While this is currently a manufacturer-specific paradigm, similar scoring systems exist for other available bioimpedance devices. BIS is recognized for its non invasive effectiveness in measuring extracellular fluid and detecting subclinical changes indicative of lymphedema onset [42, 47]. It offers high sensitivity, standardized cut-off measurements, and excellent inter-observer variability [48, 49]. Moreover, BIS can assess intra and extracellular fluid as well as total body water [50], and is now eligible for insurance reimbursement in the United States.

Stand-on devices are available for BIS and MFBIA which incorporate stainless steel contact electrodes within hand and foot plates, with values graphically displayed over time. At this time, BIS has a larger body of research, as well as greater clinical penetration, however the extent to which BIS or MFBIA may be more advantageous than the other is unknown. Although BIS and MFBIA offer fluid and body composition measurements and are relatively quick to use, there is an expense to obtaining the technology which may also be on a subscription basis, and the information obtained in the severely obese population remains questionable [51].

#### Tissue dielectric constant—percent water content

Tissue dielectric constant (TDC) are focal measures of skin-to-fat water providing a quantified measure of tissue water within the skin and subcutis to aid a clinician’s assessment of local edema detection [52]. Advantages to TDC is its portable and focal assessment of superficial tissue water content for suspicious tissue allowing for comparison to contralateral focal body region. Focal measurements can be obtained on any skin region where newer devices provide user feedback on force and have the option of calculating the percent water ratio to “spot” scan for impairment. This portable tool provides rapid localized information on superficial water content on any skin region including torso, breast, top of hand and foot, and head and neck. TDC readings can be converted to percentage water content (PWC) for ease with comparing involved or at-risk with contralateral body regions [53]. Growing evidence reveals its use in early detection of breast lymphedema and the use of percent water content [52–57]. Disadvantages include the analysis of fluid is superficial to the skin and subdermis, depth assessment varies with the probe used typically ranging from 0.5 to 5.0 mm, focal measurement does not allow for efficient whole limb surveillance for edema, normative values appear to vary across body regions and are sparsely reported to-date, and evidence is lacking in how measures may change with advancing stages of lymphedema. Though this device also cannot be used without clinical examination, it offers promising use for focal swelling and comparison of contralateral sites on the trunk, breast, head, neck, feet and hands to provide clinicians an objective noninvasive measure of superficial fluid levels.

#### Tonometry

Tissue tonometry evaluates the resistance to pressure exerted on the skin providing an output of tissue induration in Newtons (N). Measurements assess local tissue areas and newer devices provide user feedback on speed and pressure for measurement accuracy. Some models require repeat measures and automatically compute the measurement average. The local skin measurement takes less than one minute to assess for an experienced clinician using the tool. Advantages to tonometry device are its ability to provide a measurement of tissue firmness and its portability and inter reliability [58]. Disadvantages are that measures may be influenced by ambient fluctuations, there are challenges with repeat measurement, device expense, limited literature on normative and impaired values, and uncertainty as to how the measures will vary across the stages of lymphedema [35, 58], as fibrotic areas may soften with treatment while overly soft and edematous regions may harden with treatment. Research suggests potential for tonometry to inform on tissue fibrosis and treatment efficacy [59–62]. Further research is essential for a more in-depth understanding of this technology’s utility toward clinical assessment and treatment allocation in lymphedema.

#### Three-dimensional (3D) imaging

In the setting of lymphedema, 3D imaging technology advancements offer fast, noninvasive anthropometric body measurements of volume and circumference for baseline and longitudinal uses. The technology may involve scanners or a camera sensor attachment to a device with software subscription to obtain the images, in which currently limb and torso measurements are the most commonly measured. There is also the option for measurements of the hands, feet along with potential for the head and neck, necessitating further research to optimize the options across all body regions for clinical use. The 3D imaging outputs typically involve a silhouette of the body image captured providing total volume and circumferential measurements currently up to every 4 mm along the torso and limbs (arms and legs). The speed of obtaining circumferential and volumetric measures through 3D imaging offer a fast and portable opportunity for clinics to obtain a substantial amount of anthropometric data illustrating the change of volume and circumference over time [63, 64]. Though this technology is efficient and reliable for certain body regions to use for the trained user, ongoing research is necessary to optimize model fitting and measurement extraction for entire body regions. Compared to the tape measure, the technology offers more promise with speed and interrater accuracy, yet this technology is more costly potentially involving a software subscription. Similar to perometry, 3D imaging currently cannot provide proficient data for “tight” circumferential measurements needed in particular regions when fitting for custom compression garments.

#### Patient reported outcome measures

Patient reported outcome measures (PROMs) assess latent constructs such as symptoms, quality of life (QoL), and self-efficacy that could not be otherwise quantified. PROMs typically measure defining, yet subjective, dimensions of a patient’s experience, and may be condition-specific or generic. In the case of lymphedema-specific PROMs, tools have been developed and validated to evaluate the impact of lymphedema on the physical, psychoemotional, functional, and social aspects of a patient’s life [65–70]. Most lymphedema-specific PROMs have been characterized with respect to both their psychometric (e.g., Cronbach’s alpha, intraclass correlation coefficients, etc.) and general measurement properties (e.g., reliability, responsiveness, validity, etc.) [71, 72]. Access to performance information becomes critical when selecting contextually appropriate PROMs for clinical and investigative applications, since instruments may perform inconsistently across subgroups defined by lymphedema location, severity, and etiology, as well as in the presence of comorbid conditions like obesity and organ failure [73].

A subset of lymphedema-specific PROMs has been evaluated as screening tools to detect lymphedema. Separate instruments are used to screen for upper and lower extremity lymphedema. Upper extremity instruments include the Lymphedema Breast Cancer Questionnaire (area under the curve, or AUC, not reported) [74, 75], and a telephone screening questionnaire developed by Norman et al. [76](sensitivity 0.96 and specificity 0.75). Lower extremity instruments include the Lower Extremity Lymphedema (LEL) Screening Questionnaire (AUC of 0.92) [73] and the Gynecological Cancer Lymphedema Questionnaire (GCLQ) (AUC of 0.95) [77].

Generic, or condition agnostic, PROMs have been shown to have acceptable to excellent measurement properties in the assessment of function and QoL among patients with lymphedema [78, 79]^.^ Use of generic PROMs allows for cross population comparisons and use of PROM data collected by other clinical disciplines. Additionally, generic PROMs have been developed and validated for administration via computerized adaptive testing (CAT) to increase precision and efficiency. At present, only two lymphedema-specific PROMs are available for CAT administration [80, 81].

As lymphedema assessment tools PROMs offer salient benefits including low cost, ease of repeated longitudinal assessment, simple scoring and interpretation, availability in diverse languages, remote administration, and non-necessity of specialized training or personnel for high fidelity assessment. PROMs offer the only standardized means of quantifying the impact of lymphedema most pertinent to the patient, its treatment, and sequelae on a patient’s lived experience. However, PROMs are also subject to important limitations including respondent burden, inconsistent correlation with objective measures of lymphedema severity [82], and biases such as social acceptability, central tendency, end aversion, among others. Additionally, they may perform inconsistently across patient subgroups defined by sociodemographic and clinical characteristics.

### Advanced imaging measures

#### Lymphoscintigraphy

**Fig. 1:**
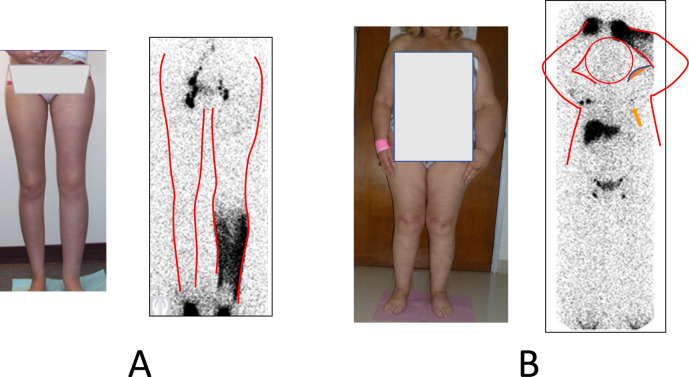
Lymphoscintigraphy for assessment of lymphatic function. **a** Lower extremity lymphedema showing pooling of lymphatic fluid in left calf and decreased left pelvic lymph node activity. **b** Postmastectomy lymphedema with pooling in distal left upper limb and markedly reduced axillary lymph node activity

Lymphoscintigraphy has been considered the primary investigation to confirm the diagnosis of lymphedema, visualizing the functional status of the lymphatic system and guiding the management of lymphedema patients [83]. Lymphoscintigraphy is accurate and does not risk allergic reactions to contrast dye [84, 85]. Lymphoscintigraphy has a sensitivity of 97% and a specificity of 100% [86]. The test requires injecting technetium Tc99m labeled antimony sulfur or albumin into the affected extremity’s second interdigital web space. Sequential images of bilateral extremities are performed in 20 min, and 3 h [87]. Lymphatic dysfunction is diagnosed if lymphoscintigraphy exhibits delayed transit time of the radiolabeled colloid to the regional lymph nodes, dermal backflow, asymmetric node uptake, and/or formation of collateral lymphatic channels [88, 89] (Fig. 1). Lymphoscintigraphic study of breast cancer-related lymphedema has found excellent intra-rater reliability (ICC = 0.946) and interrater reliability (ICC = 0.846) [90]. Limitations include expense, planar images with limited spatial resolution (unless combined with CT), fuzzy or grainy images, radioactivity exposure and disposal issues, and time required for testing.

A combined scintigraphic and CT technique, SPECT-CT, allows functional visualization of altered lymphatic flow in three dimensions as opposed to lymphoscintigraphy which is planar, and SPECT-CT simultaneously demonstrates lymphatic stigmatae per CT imaging as above. While found to have some advantages in accuracy of lymphoscintigraphic staging of lymphedema compared to lymphoscintigraphy alone [91], it is costly and not routinely used.

#### Ultrasound

**Fig. 2:**
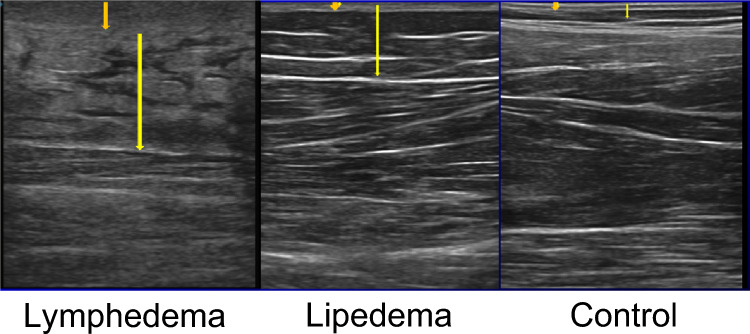
Ultrasound to distinguish normal, lymphedema and lipedema. Lymphedema is associated with increased dermal thickness and with subcutaneous tissue hyperechogenicity, whereas lipedema is associated with increased thickness and hypoechogenicity of the subcutaneous fat (dermis marked in upper arrows, subcutaneous tissues in lower arrows)

Because the physical examination is primarily subjective and does not represent subcutaneous tissue distribution and lymphedema tissue alteration, use of ultrasound has been advocated to enhance lymphedema staging [92]. (Fig. 2) A noninvasive tool, ultrasound offers visualization of the skin and subcutaneous tissue changes in extremities with chronic lymphedema, caused by changes in the extracellular matrix, such as connective tissue hypertrophy, fat accumulation resulting from both fat hypertrophy and an increased number of adipocytes, and interstitial protein-rich fluid accumulation [93]. The observation of these changes in various parts of the extremity may further elucidate the severity and extent of the disease.

Skin thickness, subcutaneous tissue thickness, and subcutaneous echogenicity all show a significant positive correlation with the ISL stage [94]. Ultrasound studies report interrater reliability, accuracy, and variability with some bias in small sample size groups.

In a study of ultrahigh frequency ultrasound of upper and lower limbs affected by secondary lymphedema, Bianchi et al. found a high correlation between the ultrasound and histological findings when used prospectively for preoperative and intraoperative analysis for lymphovenous anastomosis [95–97].

Unlike most forms of imaging, ultrasound can be administered at point of care, though user expertise is required, along with investment in the device.

#### Computed tomography

CT scans are essential in the evaluation of the causes of limb swelling. CT imaging can provide objective volume measurements and information about the structural characteristics of subcutaneous tissue in lymphedema [98]. CT scans demonstrate the alterations in epidermal and subcutaneous tissue, but can also detect neoplasms obstructing the lymphatics causing secondary lymphedema [99]. MRI and CT are useful tools for measuring both the volume of subcutaneous tissue and structural changes. CT imaging has been shown to have high sensitivity (93%) and specificity (100%) in confirming the diagnosis of lymphedema. CT imaging is not routinely applied to evaluate lymphedema because of the associated high cost and radiation exposure.

The subcutaneous tissue of lymphedema exhibits a pathognomonic honeycomb distribution of edema due to fibrotic tissue and fluid surrounding the accumulation of fatty tissue; the skin is thickened [99–101]. CT also can display the size and the number of lymph nodes, which helps define the type of primary lymphedema.

#### Lymphatic imaging with near-infrared (NIR) fluorescence

Two very similar methods for imaging shallow lymphatics use near-infrared fluorescence: Indocyanine Green Lymphography (ICG-L) and Near-InfraRed Fluorescence Lymphatic Imaging (NIRF-LI). Both ICG-L and NIRF-LI use intradermally injected ICG as a contrast dye to allow visualization of dermal lymphatics and lymph nodes to depths of 1 cm (ICG-L) or 3–4 cm (NIRF-LI) [102, 103]. Slightly different optics technology also allows NIRF-LI to image lymphatic pumping in near-real time for extended periods of time [104]. Both technologies take advantage of the fact that light in the near-infrared (NIR) portion of the light spectrum (wavelength 600–800 nm) is not readily absorbed by melanin, water, hemoglobin, or other components of human tissue. For example, if you shine a flashlight at the base of a finger, most light wavelengths will be absorbed, but NIR wavelengths will penetrate the skin between your fingers and appear red. When injected intradermally, ICG binds to intracellular albumin and other large proteins that are selectively taken up by lymphatic, but not arterial or venular, capillaries. Dim NIR laser light shining on skin excites ICG, so that shallow lymphatic vessel anatomy (and pumping, with NIRF-LI) are visible.

**Fig. 3:**
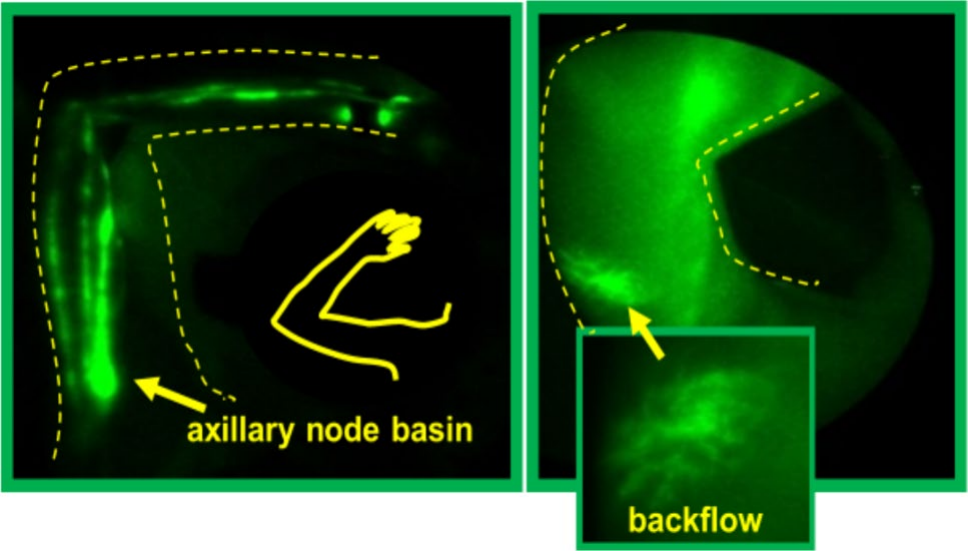
Breast Cancer-Related Lymphedema seen by Near-Infrared Fluorescence Lymphatic Imaging (NIRF-LI). Example images of (left) healthy lymphatics with well defined, linear lymphatic structure and contractile function, and (right) diseased lymphatics with fluorescent network of tortuous lymphatic vessels and dermal backflow

NIR tools can provide information as to which collector vessels are used, as well as identify anatomical areas where lymph is stagnant, appearing as “dermal backflow (Fig. 3) Dermal backflow never appears in healthy limbs, but is almost always present with subclinical and clinical lymphedema [105]. In healthy lymphatics, ICG bound to large proteins is readily transported from interstitial spaces through primary capillaries directly into lymphatic collectors. Dermal backflow in patients with lymphedema, readily detected by ICG-L, appears as cloudiness, tiny tortuous vessels clusters, or punctate bright spots within cloudy areas. Such images represent lymph that is stagnating in interstitial spaces, primary capillaries, or small pre-collector vessels.

**Fig. 4:**
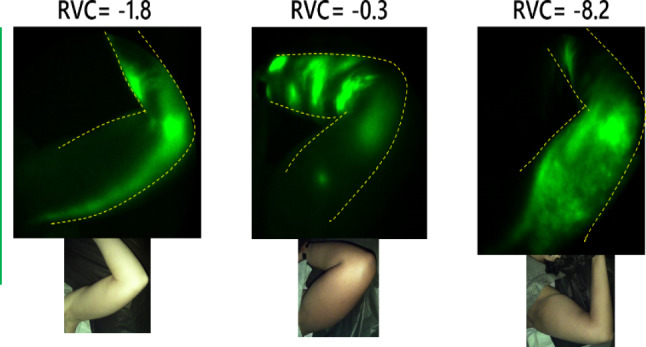
Breast Cancer-Related Lymphedema seen by Near-Infrared Fluorescence Lymphatic Imaging (NIRF-LI) in the setting of normal arm volumes. Dermal backflow (top images) is evident in three different breast cancer patients’ affected arms. White-light (bottom) images were also collected with a white-light camera mounted on the imager arm. Relative volume changes (RVC) for all three patients were negative, well below 5-10% used to clinically diagnose breast cancer-related lymphedema

These imaging modalities can be used as tools for (1) early diagnosis of lymphedema, (2) objective assessment of lymphedema conservative and surgical treatments, and (3) discernment of lymphedema in a variety of clinical contexts, not limited to post-surgical lymphedema. ICG-L and NIRF-LI can detect failing lymphatic function well before clinically recognized lymphedema can be diagnosed. Of note, lymphatic dysfunction can be missed using clinical swelling as a criterion, thus delaying treatment. For example, Fig. 4 shows NIRF-LI images of breast cancer patients clearly displaying dermal backflow, yet arm swelling is below 5% (and even negative) [106]. Such patients are assumed to not have lymphedema, and are not treated when the lymphatics may be most amenable to favorable outcomes, before irreversible tissue changes, such as skin fibrosis and subdermal adipose accumulation, are established [107]. Importantly, a recent prospective longitudinal study of breast cancer patients showed that NIRF-LI detected dermal backflow 8–23 months before clinical lymphedema was diagnosed by arm swelling [108]. NIRF-LI has shown that manual lymphatic drainage and pneumatic compression therapy directly improve lymph movement, and a study of reparative lymphatic microsurgeries is underway [109–112]. Such studies demonstrating physiologic benefits of these treatment methods have provided evidence to medical insurers in support of their reimbursement. NIRF-LI has detected dermal backflow in breast cancer patients who have yet to receive mastectomy or lumpectomy with axillary lymph node dissection, suggesting effects of the cancer itself, [108] of neoadjuvant chemotherapy [113] and/or effects of inflammation (which has been shown to systemically halt lymph pumping) [114], as risk factors for lymphedema. Breast cancer patients who receive immediate lymphatic repair at the time of axillary lymph node dissection (the lymphatic microsurgical preventative healing approach, or LYMPHA technique) are reported to have lower incidence of lymphedema [115]. Longitudinal NIR imaging could track efficacy of LYMPHA, to determine if lymphatic pumping is always restored with lymphatic-vein anastomosis.

A major limitation of ICG-L and NIRF-LI is the inability to show deep lymphatic vessels, such as the cisterna chyli/thoracic duct, although NIRF-LI can detect pulsing in collector vessels that are 3–4 cm deep, if there is no dermal backflow overlaid [103]. Nonetheless, in several cases, NIRF-LI has detected dermal backflow in patients with normal lymphoscintigraphy results, allowing early treatment for extremity lymphedema [102].

### MRI

**Fig. 5:**
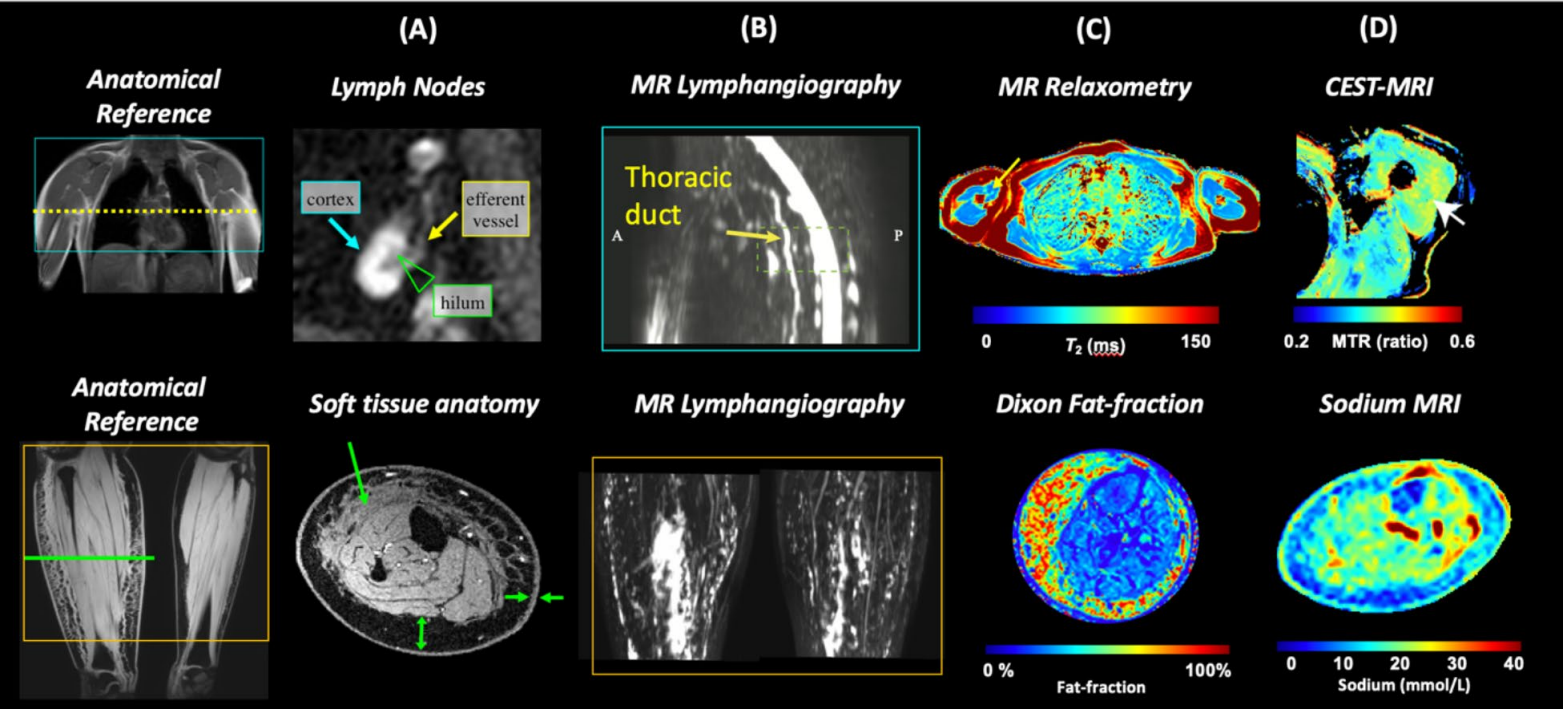
Noninvasive MRI methods for structural and physiologic lymphedema assessment. Noninvasive MRI methods provide structural and physiologic imaging measures for upper-extremity lymphedema (top row) and lower-extremity lymphedema (bottom row). **A** Optimized image contrast at 3T MRI provided lymph node anatomical imaging sensitive to the afferent and efferent vessel and lymph node substructures. Structural MRI of soft tissue anatomy demonstrates tissue remodeling in disease such as skin and adipose tissue thickening, and fibrosis deposition. **B** MR lymphangiography visualizes deep lymphatic vessel morphology, such as in the thoracic duct anterior to the spinal cord. Edema is also visualized as hyper-intense signal on non-tracer MR lymphangiography to localize dependent edema and response to manual therapies. **C** Tissue composition in lymphedema can be quantified with 3T MR relaxometry (e.g. T_2_ relaxation time map in upper-extremity unilateral lymphedema), and Dixon fat-fraction mapping. These measures indicate edematous and heterogeneous tissue in lymphedema, which responds to manual lymphatic drainage therapy. **D** Molecular imaging methods relevant for lymphedema include CEST-MRI of proteins (e.g. magnetization transfer ratio, MTR of amide proteins) and sodium MRI of endogenous tissue sodium content (mmol/L). Molecular imaging is being investigated for early biomarkers of edema formation and objective, localized measures of lymphatic disease severity. Together, MRI is a versatile modality to investigate lymphatic physiology and disease with high potential for clinical translation.

Magnetic resonance imaging (MRI) is a biomedical imaging modality with versatile image contrast that can be used to visualize anatomy at high spatial resolution, quantify tissue molecular composition, and probe vascular morphology and function in the lymphatic network comprised of vessels, nodes, and organs. MRI methods are clinically feasible to perform anatomical imaging at 3D high spatial resolution (typically 0.5–1 mm^2^ in-plane) by standard T_1_- and T_2_-weighted MRI. Anatomical imaging in lymphedema can be used to measure the limb’s volume and soft tissue remodeling such as skin or subcutaneous adipose tissue thickening, for instance (Fig. 5). MRI contrast can also be sensitized to tissue molecular content in lymphedema. Dixon MRI separately visualizes water- and fat-dominant tissue composition in a single acquisition, and can quantify tissue water and fat-fraction asymmetries to inform disease severity and location [116, 117]. MR lymphangiography with or without exogenous contrast agents is also clinically feasible with standard pulse sequences available on most hospital scanners, although further standardization of protocols and tracers is needed [118]. Non-tracer MR lymphangiography hyper-intense signal patterns in subcutaneous edema and tissue sodium content by sodium MRI are being investigated to inform individualized physical therapy strategies for edema mobilization [119, 120]. While current cost and accessibility limit the widespread use of MRI in clinical lymphedema management, its high spatial resolution, noninvasive molecular specificity, and adaptability to functional lymphatic imaging offer particular advantages and clinical use scenarios [121].

MRI offers exciting possibilities as a research tool to advance lymphedema care. Examples of advancements in lymphatic MRI include MR relaxometry of lymphatic fluid, lymph nodes, and soft tissue in lymphedema that is fundamental to optimizing anatomical image contrast across the lymphatic system [122–124]. MR lymphangiography has advanced in specificity for lymphatics by combining T_1_ and T_2_ shortening agents with techniques like DARC-MRL and contrast-enhanced MRL [125]. Dynamic lymphangiography analysis tools will be needed to translate these techniques into a clinical setting. Physiologic imaging is exploring MRI quantification of tissue protein, sodium, and fat composition in lymphedematous tissue to investigate the impact of lymphatic dysfunction on limb physiology and potentially image-based personalized risk factors for developing lymphedema or lipedema [126–128]. As novel therapeutics are developed for lymphedema, MRI modalities could likely play a significant role as objective, sensitive outcome measures of therapeutic mechanism and response to therapy. MRI research opportunities remain at all translational research levels to improve the specificity and objectivity of lymphatic disease assessment.

### Dysmorphism: Shape and contour deviations. Metaplasia: Altered tissue integrity that is not on malignant trajectory.

### Lymphedema evaluation: tables and summaries

No individual method is effective for the entire range of lymphedema subdomains Domains Assessed by Specific Modalities (Table 1), with the fewest options available for dysmorphism and metaplasia. H&P, PROM’s, MRI and CT show the strongest multidimensional capabilities, with ultrasound and ICG also promising. The various methods available to assess volume are largely unidimensional, however some measures (perometry, 3D camera, and to some extent tape measure) do give detail about contour. Technologies such as bioimpedance and TDC, which assess fluid content, are becoming increasingly available, but these are limited in their ability to delineate other tissue composition factors such as fibrosis or fat, which require use of advanced measures including ultrasound, CT and MRI. Measures which are both clinically feasible and have good discrimination for soft tissue factors beyond extracellular fluid assessment are a priority for future work. Clinically feasible measures of lymphatic function are also a gap area.

**Table 1:**
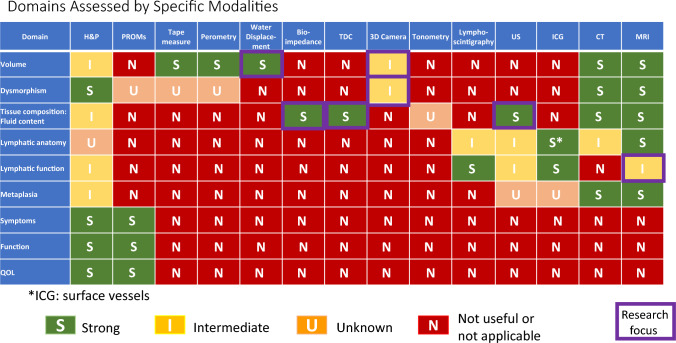
Utility of lymphedema assessment modalities for specific subdomains

(Table 2) This category relates mainly to clinical situations of individuals at risk for lymphedema, most commonly following cancer treatment. Screening constitutes an especially active focus of current lymphedema research. Furthermore, from a clinical viewpoint, mechanisms for early detection of cancer-related lymphedema are increasingly prioritized for integration into routine care [129]. The need to reconcile the dual priorities of optimizing both quality and feasibility, which are pertinent to all dimensions of lymphedema measurement, reaches primacy with screening, due to population health-level need for repeated measurements over time in a large number of patients.

**Table 2:**
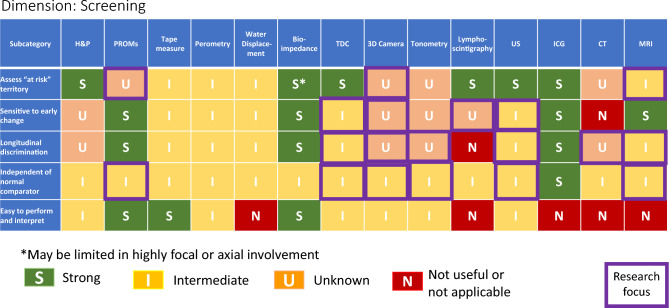
Performance of modalities in screening for lymphedema

Studies suggest that the sensitivity of bioimpedance is in the 70–76% range for cancer-related lymphedema when advanced measures including lymphoscintigraphy [48], or ICG [130, 131] are used as reference standards. Bioimpedance has also exhibited favorable specificity in some studies. [132, 134]. The international multicenter PREVENT randomized controlled trial data has found fewer patients progressing to clinically evident lymphedema when bioimpedance has been integrated into screening processes for breast cancer-related lymphedema [132]. Studies comparing bioimpedance to perometry have shown anywhere from modest [133] to good [134–136] correlation between the two modalities. Dylke et al. [48] found slightly better sensitivity and specificity with perometry (81%/96%) compared to bioimpedance spectroscopy (76%/93%), using lymphoscintigraphy as the reference standard, in the context of mild to moderate upper limb lymphedema. On the other hand, Czerniec et al. [136], while finding high concordance between bioimpedance spectroscopy and perometry, found significantly higher interlimb ratios with bioimpedance spectroscopy than perometry in individuals with clinically identified mild to severe lymphedema, and this pattern held true with both whole-arm and segmental analyses.

Overall, fluid-detection measures are becoming increasingly utilized for screening, based on favorable accuracy characteristics found in the breast cancer population, as well as feasibility, especially for Individualizing Treatment (Table 3). bioimpedance. However, this issue is not an entirely decided matter. Volumetric measurement techniques continue to be employed in screening research trials, and they also remain relevant based on common use. On the favorable side, volumetric measures exhibit value as complementary information, but on the challenging side, they can be difficult to upscale for frequent repeated measurements. Tape measurement prevails in most lymphedema practices and is particularly advantageous in stressed resource settings. Tape measurement can also be adapted for self-monitoring. Perometry has particularly good sensitivity to small volume change. 3D imaging via phone or tablet shows strong future potential given its promising convenience. PROM’s also show some favorable characteristics and, while not a stand-alone assessment, offer complementary information. In summary the use of a comprehensive multi-modal assessment is recommended with clinical evaluation and symptom reporting also important.

**Table 3:**
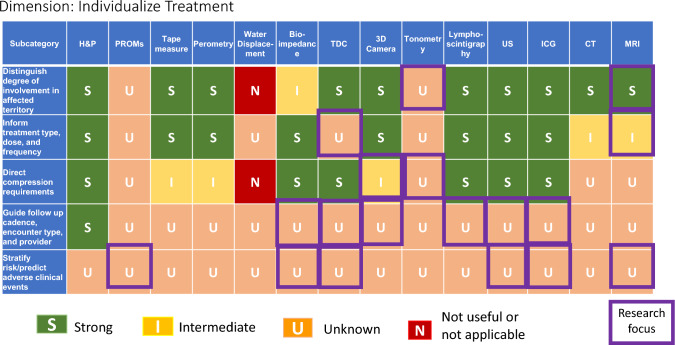
Performance of lymphedema diagnostic modalities in guiding individualization of treatement

Despite favorable aspects of bioimpedance, current technology has limits, especially with regards to detecting axial (i.e., head and neck, trunk, breast, chest wall, genitalia) lymphedema, and for evaluation of other focality (such as dorsum feet or hands). Measures that are useful for axial lymphedema, such as ultrasound and TDC, are not yet in wide use for this purpose, and have their own feasibility considerations. While bioimpedance utilization has expanded, implementation challenges remain due to cost, as, despite its FDA approval for BIS in the United States, gaps remain in insurance coverage.

Diagnosis (Table 4) pertains to a range of clinical situations not limited to cancer-related lymphedema and may entail distinguishing lymphedema from other causes of swelling or soft tissue changes, and/or establishing the underlying disease causing lymphedema. Regarding lymphedema diagnosis, a noteworthy observation is that despite multiple available methodologies, no individual modality outperforms H&P, especially among the measurement tools available in office and at the bedside. H&P provides advantages of myriad components as well as overall pattern recognition. However, H&P can have limits diagnostically, especially in medically complex patients or those with mixed etiologies. Depending on context, distinguishing exact contributors may or may not make a difference in management, and H&P, including the crucial history portion, helps to identify when more testing is needed. ICG, MRI and lymphoscintigraphy do have areas of strength and can be considered when H&P alone provides insufficient objective and diagnostic confidence in distinguishing lymphedema from other etiologies, identifying earliest potential onset of lymphedema, and determining affected versus unaffected areas. Longitudinal assessment and/or utilizing more than one type of modality can also be helpful in verifying diagnosis in difficult or elusive cases.

**Table 4:**
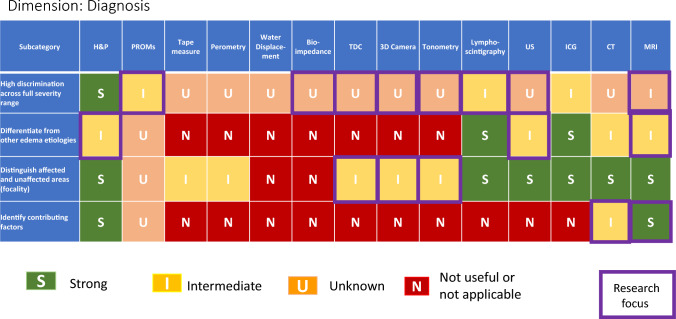
Performance of modalities in diagnosis of lymphedema

The individualizing treatment (Table 4) category shows some areas of strength, especially with regard to distinguishing degree of involvement in affected territories, and informing treatment, with H&P, ultrasound, ICG, MRI and lymphoscintigraphy showing strongest characteristics, and some advantages also seen for tape measure, perometry, bioimpedance, and TDC. Limitations remain, especially in guiding follow up logistical needs, as well as in stratifying risk for adverse outcomes, especially as risks may be affected by changes in treatments. Therefore, while useful tools exist, optimizing their integration into clinical care paradigms is a priority.

The majority of modalities are highly limited, except for Monitoring Treatment Response (Table 5). the domain of detecting volumetric changes, which shows several effective options. Of these, perometry and water displacement have the highest precision to capture change over time, though tape measure is commonly used based on feasibility. Bioimpedance has defined criteria for intervention, and favorable utility for surveillance, though the emphasis of the bioimpedance literature to-date has been towards lymphedema identification and screening rather than on its reliability in chronic situations and clinically relevant improvements during rehabilitation. Other subdomains show a lack of abundant options, especially for tissue composition and metaplasia.

**Table 5:**
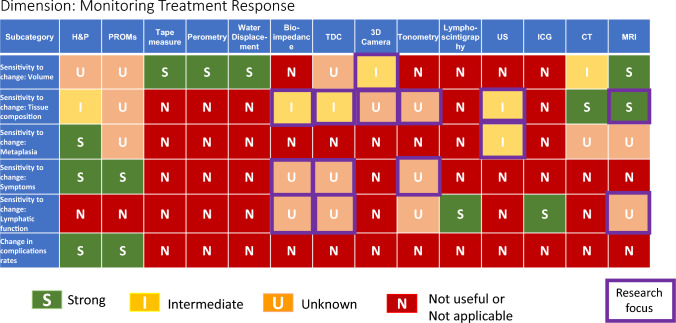
Performance of lymphedema diagnostic modalities in monitoring treatment response

## Discussion

Several concurrent approaches are layered within this work. First, a comprehensive framework has been generated of assessment dimensions and subcategories of lymphedema diagnosis and quantification, which assists with a practical understanding of the wide-ranging considerations pertinent to this diverse topic. Second the S/I/U/N ratings are assigned, employing defined criteria with emphasis on the evidence basis. Third, the grid format allows identification of overarching patterns, such as (1) the strength and weakness of particular modalities versus others within-dimensions of lymphedema evaluation (diagnosis, screening, individualizing treatment, monitoring treatment response), (2) the lymphedema characteristics which can be most confidently assessed (i.e., volume, tissue composition, symptoms, etc.), and (3) the lymphedema evaluation dimensions which overall have the strongest or weakest evidence foundation.

The overriding purpose of the above process is to produce a point-in-time snapshot of lymphedema diagnostic methods to update clinicians in their selection process of situationally appropriate modalities, to apply to their patient care as well as program development. Accomplishing this goal requires the dual priorities of highlighting traditional methods of continuing relevance, as well as areas of exciting progress. The substantial remaining gaps are also accentuated.

While specific clinical context ultimately guides decision-making, patterns seen among the various forms of lymphedema evaluation are noteworthy. Of the lymphedema domains assessed by specific modalities (as seen in Table 1), the overall highest performing modalities across the spectrum of domains, with the greatest number of strong ratings, are MRI, H&P, CT, and PROMs; the lymphedema domains most likely to have strongly rated assessment methods are volume and fluid content. Of the various dimensions of lymphedema assessment (Tables 2–5), individualization of treatment attains the highest frequency of strong ratings (45%), followed by screening (24%), diagnosis (20%), and monitoring treatment response (15%). Across the 20 subcategories within these four tables of lymphedema dimensions, H&P demonstrates the highest number of strong ratings (11), followed by ICG (10), MRI (8), lymphoscintigraphy (7), bioimpedance (6), PROMs (5), ultrasound (5), tape measure (5), perometry (3), TDC (3), CT (3), 3D camera (2), water displacement (1), and tonometry (0). When modalities receiving either strong or intermediate ratings are considered, across these 20 subcategories, the values become, in order of frequency, H&P (15), MRI (14), ultrasound (12), ICG (11), tape measure (10), perometry (9), bioimpedance (9), TDC (9), lymphoscintigraphy (8),CT (8), PROMs (7), 3D camera (7), water displacement (5), and tonometry (3).

Dysmorphism: Shape and contour deviations.

Metaplasia: Altered tissue integrity that is not on malignant trajectory.

Of note, in considering diagnostic modalities, we elected not to include simple lymphography, which has become less favored due risks of lymphatic vessel cannulation and to potential for lymphatic damage with radioopaque contrast; however, lymphography may be especially useful, for deep/central lymphatic system imaging (thoracic duct, cisterna chyli), and is considered when other advanced imaging approaches are not available [137, 138]. In the United States, ultrasound, ICG-L, MRI/MRL, CT or lymphoscintigraphy are more commonly used. But even the use of lymphoscintigraphy, considered the gold standard to diagnose lymphedema, is only used for ~ 6.7–9.5% of cancer-related lymphedema patients [139].

### Clinical vs research considerations

No single type of measure is applicable to all aspects of clinical and research integration. For routine clinical use, modalities which are feasibly integrated into office or bedside settings will be favored, with radiologic imaging measures applied more selectively. Water displacement, while cumbersome for routine clinical use, has been advocated for research given its high precision, as has perometry. PROMs show favorable characteristics for use in clinical settings, including that they capture factors such as symptoms and function which are missed by other tools and provide clinicians with patient-specific impairments to tailor personalized care. But patient burden must be considered with PROMs, as well as clinic workflow and ability to clinically incorporate the information, therefore PROM tools need to be chosen very intentionally to assess target domains as efficiently as possible. In the research setting, use of more expanded PROM item banks may be appropriate. Bedside or clinic options which constitute a particularly strong focus of current research include bioimpedance, TDC, and 3D camera. Tools such as US and ICG have not yet enjoyed widespread clinical application in diagnosis, but ultrasound is one of the stronger noninvasive, portable modalities for evaluating soft tissues, and has potential for expanded role at the bedside. Additionally, ICG is beginning to show application beyond surgical planning, such as prognostication and possible role in guiding tailored lymphedema therapy [137] MRI is a multifaceted and evolving topic in current lymphedema research that will likely have a range of future applications albeit an inherent limitation for patients not compatible for MRI use. More specific qualities can also drive selection, for example the utility of MRI and CT in characterizing lymphedema involvement at the level of deep structures.

In clinical care, cost effectiveness remains a crucial component of feasibility. However, from another vantage point, lymphedema as a diagnostic entity has been widely considered as underrecognized and undertreated. Furthermore, the role of the full range of diagnostic options should be weighed for appropriate best practice. Challenging circumstances may require the incremental precision obtainable with overlapping or hierarchical approaches. The cost benefit of additional testing will likely vary based on context. Lipedema is an example of a clinical situation where additional testing may promote cost effectiveness, sparing costly lymphedema treatment.

An imaging toolbox that utilizes several different modalities could allow optimal understanding of lymphatic dysfunction but may be costly and present accessibility difficulties. For example, to present figures at the time of this writing, ICG-L imagers are commercially available, but they can cost hundreds of thousands of US dollars, and ICG costs $110 per vial in the United States. The billing code used by reparative microsurgeons for ICG-L prior to lymphovenous bypass and/or vascularized lymph node transplant reimburses at ~ $700. ICG-L is used in Europe for assessing lymphedema treatment efficacy, but there may currently be patent issues for such use in the US. NIRF-LI is only available for research use at present. Comparatively, lymphoscintigraphy averages $1500, and MRL may cost $900. Cost effectiveness studies are needed, with an imaging protocol streamlined to maximize the cost/benefit ratio.

### Current and emerging themes

1. Simultaneous with judicious use of emerging technologies, traditional evaluation methods including clinical history and physical examination and other office-based measures remain highly relevant mainstays of comprehensive assessment for theexperienced clinician, who can order and interpret correctly the various modalities described.
2. Specific practice settings, especially whether high, medium or low in resources will influence feasibility in translation of available techniques.
3. Need exists for improved bedside measures of soft tissue characteristics, including tissue composition (moving beyond fluid content), dysmorphism and metaplasia.
4. Bioimpedance and PROMs may be particularly valuable for early detection and surveillance of lymphedema.
5. TDC is showing strong potential for detecting and assessing focal lymphedema.
6. 3D camera is another emerging tool, for which more research is needed, with potential advantages including speed (compared to tape measurement) and feasibility factors (compared to perometry). 3D camera is also showing favorable spatial resolution (4 mm).
7. Use of ultrasound holds promise for more widespread use in evaluating tissue characteristics, especially for focal lymphedema, and has potential to serve as a bridge between bedside availability and traditional diagnostic imaging.
8. ICG and MRI to-date have been employed mainly in specialized situations such as presurgical evaluations and research, however potential clinical applicability has far-reaching implications, including guaging the effectiveness of therapy approaches on a physiological level.
9. Additional research studies focusing on correlation between advanced modalities (such as MRI, ICG or lymphoscintigraphy), and bedside tools such as bioimpedance, TDC, and perometry are encouraged to better inform clinical practice.
10. Imaging techniques such as MRI have a role in development of new therapies such as mechanistic or functional based treatment, even if not easily translatable into routine clinical care.
11. Application of artificial intelligence (AI) has potential to assist with interpretability of lymphedema diagnostic testing. However, a need exists for more standardized acquisitions with techniques to allow AI systems to be more reliable.

### Limitations

In formulating the above charts and assigning specific ratings, the authors acknowledge inherent interpretative limitations due to (1) heterogeneous clinical contexts, including patient mix, physical, financial and workforce resources of various care environments, and models of care, and (2) varying technologies within some of the modality categories. Because of this contextual variance, our expert panel employed “current usual circumstances” as a best-fit guiding principle in gauging a particular modality, and also assumed that end-user clinicians or researchers were appropriately trained and experienced in use of the particular modality. However, even with these assumptions, the authors emphasize that the focus for the reader is most appropriately placed on patterns of observations, rather than considering any one particular rating as definitive for all situations.

Additionally, there have been some limitations in focus, most notably that this work has emphasized peripheral lymphedema, rather than the central lymphatic system. The lymphatic system is highly heterogeneous with peripheral and central aspects. Internally, the lymphatic networks will vary according to whether the topic is hepatic, intestinal, cardiac, and so forth. [137] Central lymphedema evaluation may center around evaluating for structural disruptions such as fistula or leakage [140], whereas peripheral etiologies are more likely to relate to lymphatic congestion [137], with the latter being implicit in this paper. Head and neck lymphedema may include external or internal components, or both. [141] In the brain, a combination of glyphatic system (parenchymal) and lymphatic (meningeal) function has been found [142].

Various other limitations are present. Much of the literature examining office-based modalities is problematic with regard to reference standards, obscuring the ability to interpret sensitivity, specificity and other aspects of empirical data. The literature overall is skewed towards breast cancer-related lymphedema; while this emphasis is understandable given its prevalence and the potential to intervene early, other clinical contexts, including lower limb etiologies, merit more attention. Some important related aspects are beyond the scope of this paper, including surgical planning, surveillance time-course models, and clinical aspects of risk factor evaluation. Laboratory medicine aspects of lymphedema such as genetic, biomarker, or inflammatory aspects of lymphedema are not explored. While cost factors are given consideration in our discussions of the modalities, detailed cost effectiveness analyses are beyond the current scope of available evidence Lastly, while we attempted to highlight areas particularly relevant to research, we acknowledge and even emphasize that virtually all of the domains and modalities assessed in this paper are relevant to lymphedema research.

## Conclusions

Lymphedema diagnostic and quantitative methods encompass an increasing array of modalities, which are pertinent to various lymphedema characteristics, and applicable to clinical and research aims, including dimensions of diagnosis, screening, individualization of patient care, and monitoring treatment response. This paper provides guidance to clinicians in (1) affirming the role of traditional measures when appropriate, and (2) identifying instances when integrating emerging technologies and imaging modalities can enhance patient care.

## Data Availability

No datasets were generated or analysed during the current study.
